# Diagnostic value of fetal hemoglobin Bart’s for evaluation of fetal α-thalassemia syndromes: application to prenatal characterization of fetal anemia caused by undiagnosed α-hemoglobinopathy

**DOI:** 10.1186/s13023-022-02197-w

**Published:** 2022-02-10

**Authors:** Kritsada Singha, Supawadee Yamsri, Attawut Chaibunruang, Hataichanok Srivorakun, Kanokwan Sanchaisuriya, Goonnapa Fucharoen, Supan Fucharoen

**Affiliations:** 1 Centre for Research and Development of Medical Diagnostic Laboratories, Faculty of Associated Medical Sciences, Khon Kaen University, Khon Kaen, 40002 Thailand; 2 Faculty of Medicine, Mahasarakham University, Mahasarakham, Thailand

**Keywords:** Hemoglobin Bart’s, α-Thalassemia syndrome, Hemoglobin Constant Spring, Prenatal diagnosis, Fetal anemia

## Abstract

**Background:**

To evaluate whether the quantification of fetal hemoglobin (Hb) Bart’s is useful for differentiation of α-thalassemia syndromes in the fetus and to characterize the fetal anemia associated with fetal α-hemoglobinopathy.

**Methods:**

A total of 332 fetal blood specimens collected by cordocentesis were analyzed using capillary electrophoresis and the amount of Hb Bart’s was recorded. The result was evaluated against thalassemia genotypes determined based on Hb and DNA analyses. Prenatal Hb and DNA characterization of the fetal anemia observed in two families was done.

**Results:**

Among 332 fetuses investigated, Hb and DNA analyses identified 152 fetuses with normal genotypes. The remaining 180 fetuses carried α-thalassemia with several genotypes. Variable amounts of Hb Bart’s were identified in all fetuses with α-thalassemia, which could be used for simple differentiation of fetal α-thalassemia genotypes. These included α^+^- and α^0^-thalassemia traits, homozygous α^+^-thalassemia and Hb Constant Spring (CS), Hb H disease, Hb H-CS and Hb H-Quong Sze diseases, homozygous α^0^-thalassemia causing the Hb Bart’s hydrops fetalis and a remain uncharacterized α-thalassemia defect. The previously undescribed interactions of Hb Queens Park and Hb Amsterdam A1 with Hb E were detected in two fetuses with Hb Bart’s of 0.5%. The Hb Queens Park-AEBart’s disease was also noted in one pregnant woman. Prenatal analysis of the fetuses with severe fetal anemia and cardiomegaly with Hb Bart’s of 9.0% and 13.6% revealed unexpectedly the homozygous Hb CS and a compound heterozygosity of Hb CS/Hb Pakse’ with Hb E heterozygote, respectively.

**Conclusions:**

The usefulness of detecting and differentiation of fetal α-thalassemia syndromes by quantifying of Hb Bart’s was demonstrated. Apart from the fatal condition of Hb Bart’s hydrops fetalis associated with homozygous α^0^-thalassemia, homozygous Hb CS and a compound Hb CS/Hb Pakse’ could result in severe fetal anemia and fetal complications, prenatal diagnosis is highly recommended. The simple Hb Bart’s quantification of fetal blood should prove helpful in this matter.

## Introduction

α-Thalassemia is one of the most common inherited hemoglobin (Hb) disorders. In northeast Thailand, the prevalence of α-thalassemia is around 30%, consisting of both α^0^-thalassemia (5–6%) and α^+^-thalassemia (24–25%). α^0^-Thalassemia caused by a deletion of two α-globin genes (--/αα) is the most severe form which in the homozygous state can lead to a fatal condition known as Hb Bart’s hydrops fetalis. Most of the α^+^-thalassemia caused by the deletion of one α-globin gene (-α/αα) is generally mild [1, 2]. Interaction of these α-thalassemia leads to the Hb H disease, and interaction of Hb H disease with Hb E leads to complex syndromes of AEBart’s, EFBart’s, and EEBart’s diseases, with thalassemia intermedia phenotypes, commonly encountered in the region [3–6]. However, the interaction of α^0^-thalassemia with non-deletional α^+^-thalassemia and some forms of homozygous α^+^-thalassemia caused by point mutations may result in more severe clinical phenotype and unstable Hbs, requiring appropriate management, treatment, and genetic counseling [7–9]. Therefore, identification of these α-thalassemia defects is essential. Unfortunately, in Thailand and other Southeast Asian countries, the prevention and control program of thalassemia only targets at three severe thalassemia diseases, including homozygous α^0^-thalassemia, β-thalassemia major, and β-thalassemia/Hb E disease. The program aims to offer carrier screening and identify carriers of α^0^-thalassemia, β-thalassemia, and Hb E [10, 11]. Prenatal diagnosis is then offered to a couple at risk of having a fetus with the three thalassemia diseases. Other thalassemia genotypes are not included. Fetal α-thalassemia genotypes are defined by DNA analysis [12].

At our routine service for thalassemia, when prenatal diagnosis is performed on fetal blood specimen, we also carry out Hb analysis using capillary electrophoresis in addition to DNA analysis. We evaluate in this study if this Hb analysis which is relatively simpler compared to DNA analysis, is helpful in a routine prenatal diagnosis and differentiation of fetal α-thalassemia syndromes in areas with high prevalence and diverse heterogeneity of thalassemia and hemoglobinopathies.

## Materials and methods

### Subjects and hematological analyses

Ethical approval of the study protocol was obtained from the Institutional Review Board of Khon Kaen University, Khon Kaen, Thailand (HE 622173). Retrospective data at the routine thalassemia diagnostic service at the Centre for Research and Development of Medical Diagnostic Laboratories, Faculty of Associated Medical Sciences, Khon Kaen University, Thailand, were collected from a total of 332 fetal blood specimens subjected to prenatal diagnosis of α-thalassemia. The specimen was collected using the method of cordocentesis, and maternal cell contamination was monitored in each fetal specimen using the Hb F cell staining [12]. Complete blood count was performed using a standard blood cell counter. Hb analysis was performed using the capillary electrophoresis (CapillaryS 2; Sebia, Lisses, France).

### Routine DNA analysis

Common α-thalassemia found in Thailand (--^SEA^, --^THAI^, -α^3.7^, -α^4.2^, Hb Constant Spring (HBA2:c.427T>C) and Hb Pakse′ (HBA2:c.429A>T), α-globin gene triplication (ααα), Hb variants, and Hb E were identified routinely using PCR-related techniques as described elsewhere [12–15]. Hb Quong Sze mutation (HBA2: c.377T>C) on an α2-globin gene was identified by PCR–RFLP assay using *Msp*I restriction digestion (5′-C^▼^CGG-3′) as described [16]. Uncharacterized samples were further examined by direct DNA sequencing. Thalassemia genotypes were defined based on the results of these DNA analyses.

### Identification of α^Hb Amsterdam A1^ and α^Hb Queens Park^ mutations

To provide a method for rapid identification of the α^Hb Amsterdam A1^, and α^Hb Queens Park^ mutations found in this study, the PCR–RFLP assay using *Bts*CI restriction enzyme was developed. This method has been described for detection of the α^IVSI-117(G>A)^ mutation (HBA1:c96-1G>A) located in the vicinity of the two variants on α1-globin gene [6, 17]. Selective amplification of α1-globin specific fragment (975 bp) was performed by PCR using primers C1 (5′-GCCTCTTTGCACCATTCTAA-3′) and B (5′-AATGCACTGACCTCCCACAT-3′). The PCR product was digested to completion with *Bts*CI restriction enzyme (5′-GGATGNN^▼^-3′) (New England Biolabs, Beverly, MA, USA). A normal control fragment is digested into the 457 bp, and 518 bp fragments, whereas the 975 bp remain undigested fragment indicates the α^Hb Amsterdam A1^, and α^Hb Queens Park^ mutations.

### Prenatal characterization of fetal anemia and cardiomegaly

**Fig. 1:**
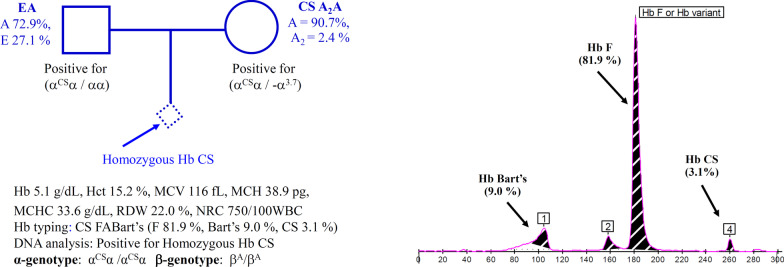
Pedigree analysis of a family in which the father was a double heterozygote for Hb E/Hb CS and the mother was a compound Hb CS/α^+^-thalassemia (3.7 kb deletion). Fetal blood analysis identified that the fetus was a homozygous Hb CS. Hematological parameters of the fetus are listed and Hb analysis profiles using capillary electrophoresis are shown with Hb Bart’s 9.0%

**Fig. 2:**
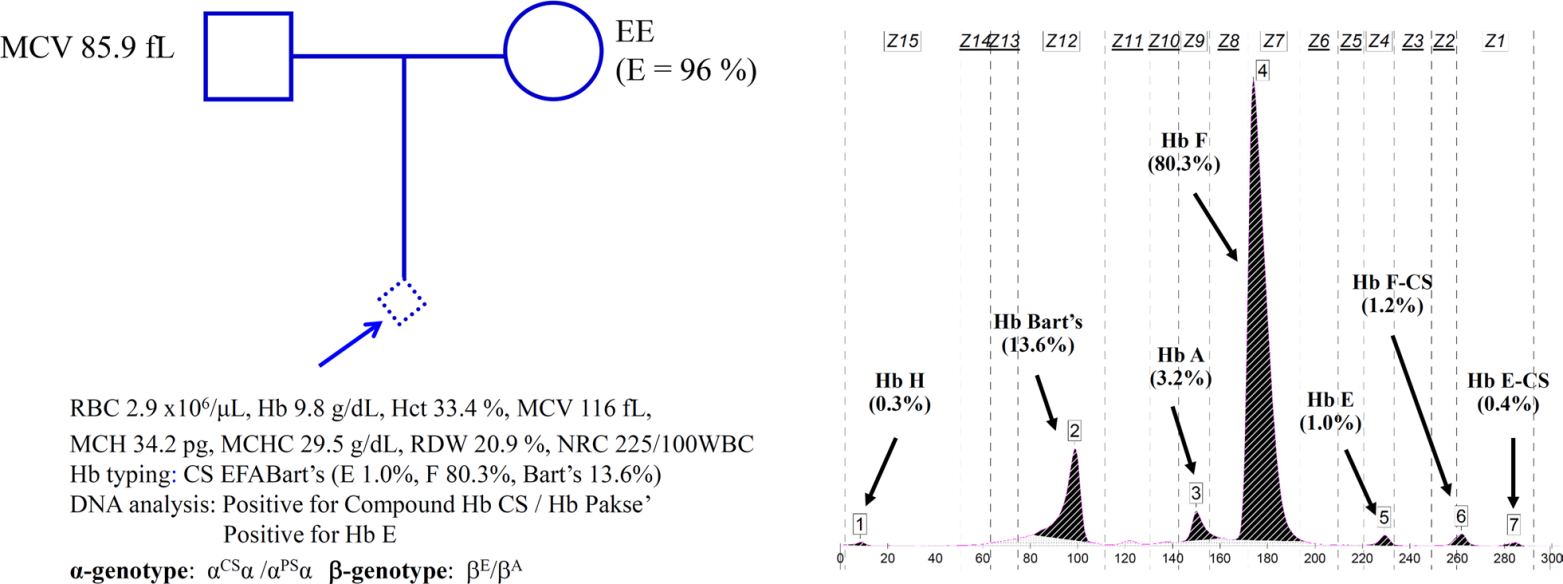
Pedigree analysis of a family in which fetal blood analysis identified that the fetus was a compound heterozygote for Hb CS/Hb Pakse’ in association with heterozygous Hb E. Due to the lack of specimens of the parents, their exact thalassemia genotypes could not be defined. Hematological parameters of the fetus are listed and Hb analysis profiles using capillary electrophoresis are shown with Hb Bart’s 13.6%

Prenatal characterization was performed on two unrelated families. In Family 1 (Fig. 1), the father was diagnosed on Hb analysis as Hb E heterozygote, whereas his pregnant wife was a carrier of Hb CS. Based on the protocol used in a prevention and control program of thalassemia in Thailand, they had no risk of having fetus with severe thalassemia disease. However, in the second trimester of pregnancy, the fetus developed fetal anemia due to an unknown cause. Fetal blood was taken by cordocentesis at a gestational age of 27 weeks and transferred to us for thalassemia investigation. In Family 2 (Fig. 2), fetal blood was taken at a gestational age of 34 weeks because of fetal anemia and fetal cardiomegaly. The mother was diagnosed based on Hb analysis as homozygous Hb E and her husband was a normal subject with MCV 85.9 fL. As for family 1, they had no risk of having fetus with severe thalassemia. The fetal blood was sent to us for thalassemia investigation.

## Results

**Fig. 3:**
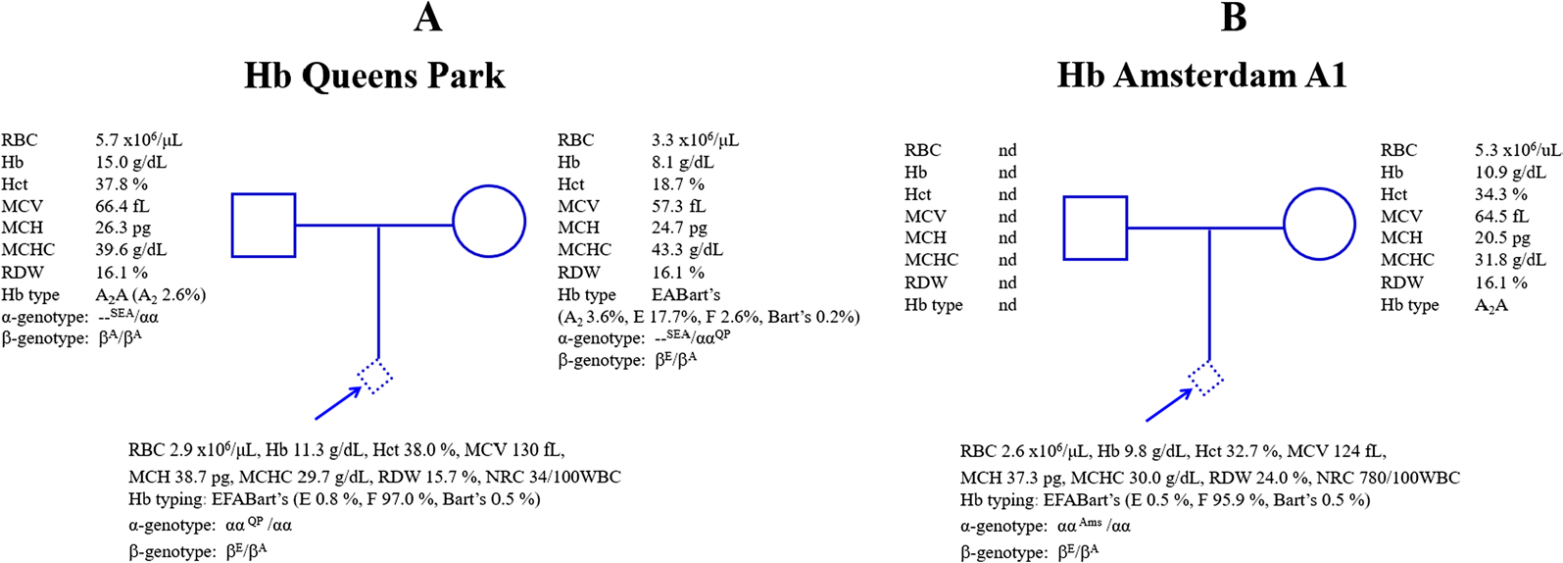
**A** Pedigree analysis of a family in which the fetus was a double heterozygote for Hb Queens Park/Hb E. The father was a carrier of α^0^-thalassemia (SEA deletion) and the mother was a patient with Hb H-Queens Park disease with Hb E heterozygote (the Hb Queens Park-AEBart’s). Hematological parameters of all members are listed. **B** Pedigree analysis of a family in which the fetus was double heterozygote for Hb Amsterdam A1/Hb E. No blood specimen of the father was available. Hematological parameters of the mother and the fetus are listed

**Fig. 4:**
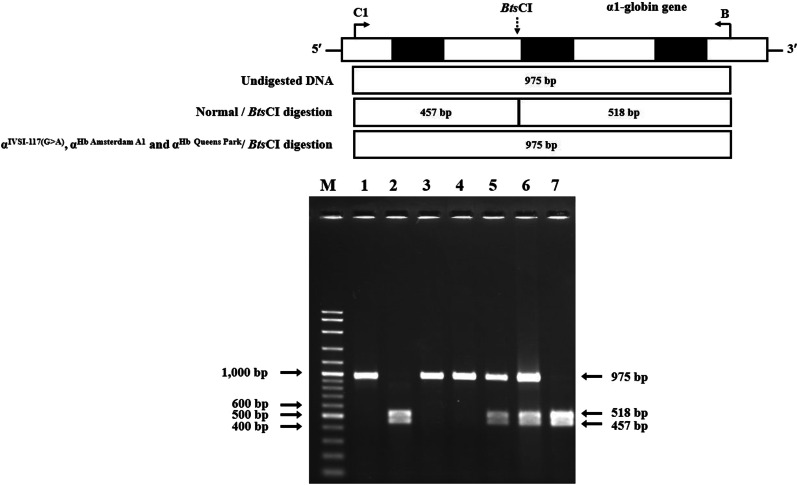
Identification of α^Hb Amsterdam A1^, α^Hb Queens Park^ and α^IVSI−117(G>A)^ mutations on α1-globin gene by PCR–RFLP assay using *Bts*CI digestion as described in the Materials and methods section. The length of the amplified fragment is 975 bp. Upon digestion, the normal allele is digested into two DNA fragments with 457 bp and 518 bp in lengths, whereas the mutant counterpart remains undigested at 975 bp in length. M represents the GeneRuler 100 bp plus DNA ladder. **1**: Undigested amplified DNA, **2**: *Bts*CI-digested amplified DNA of compound heterozygous α^0^-thalassemia and α^IVSI−117(G>A)^, **3** and **4**: *Bts*CI-digested amplified DNA of the normal subjects, **5**: *Bts*CI-digested amplified DNA of Hb Amsterdam A1 trait, **6**: *Bts*CI-digested amplified DNA of Hb Queens Park trait, and **7**: *Bts*CI-digested amplified DNA of a compound heterozygous for α^0^-thalassemia and Hb Queens Park with Hb E heterozygote (the Hb Queens Park AEBart’s disease)

**Fig. 5:**
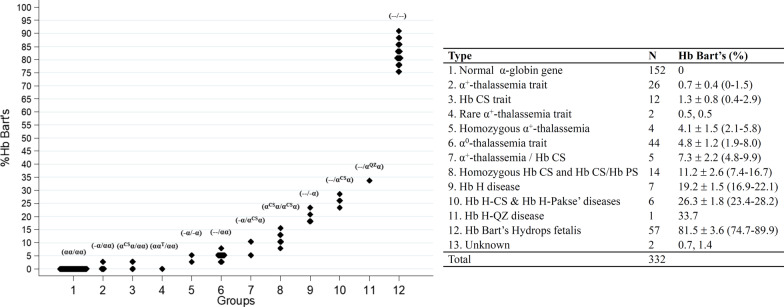
Plots of percentages of fetal Hb Bart’s identified in normal fetuses and those with various α-thalassemia syndromes. α-Genotypes based on DNA analysis and the number of cases in each genotype is indicated. Percentages of Hb Bart’s are shown as mean ± SD (range)

**Fig. 6:**
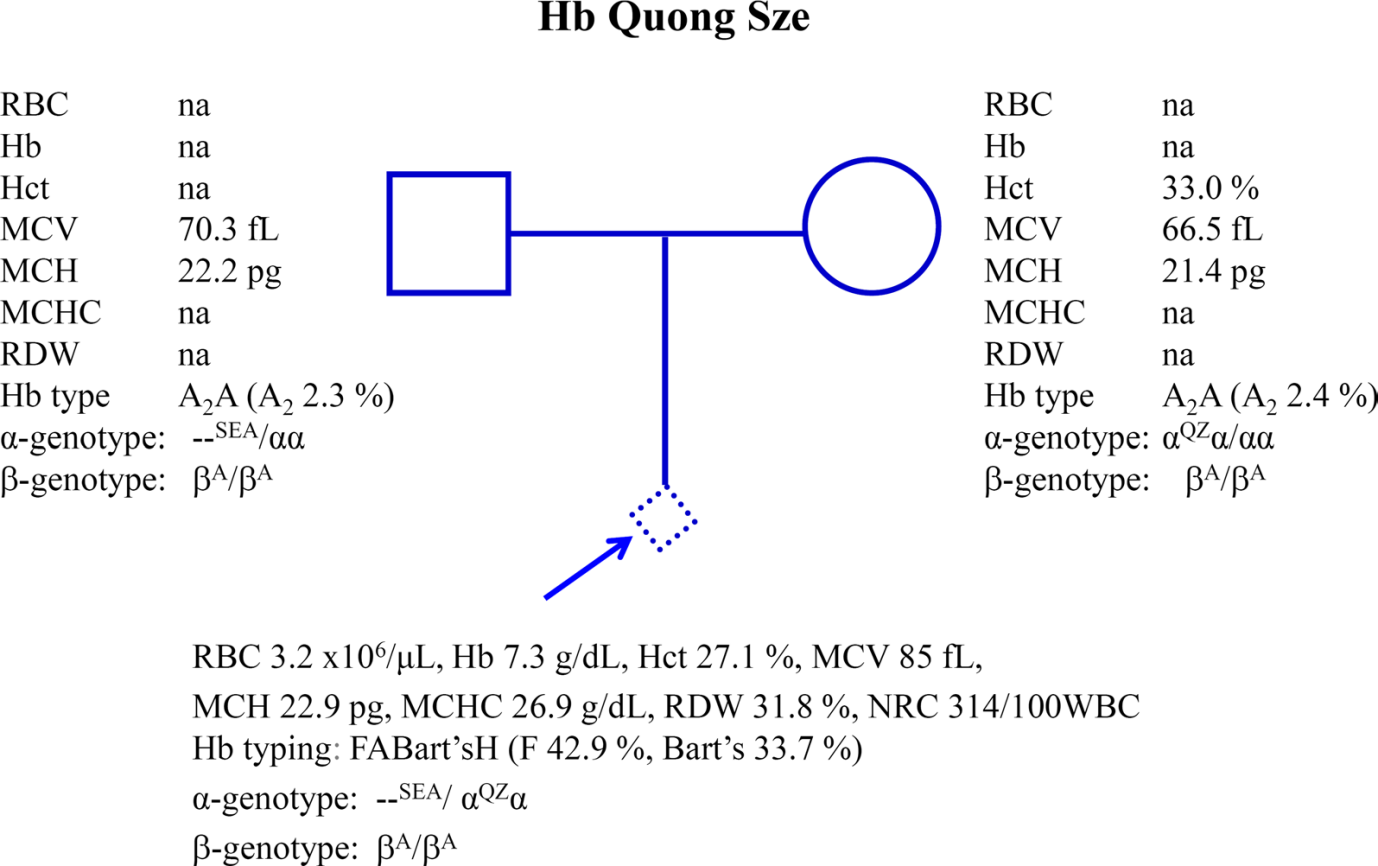
Pedigree analysis of a family in which fetal blood analysis identified that the fetus was suffered from the Hb H disease with Hb Quong Sze (--^SEA^/α^QZ^α). In contrast, the father was a carrier of α^0^-thalassemia (SEA deletion) and the mother was a carrier of Hb Quong Sze. Hematological parameters of all members are listed with the fetal Hb Bart’s of 33.7%. *na* not available

Among 332 fetuses investigated for α-thalassemia, DNA analysis revealed 152 (45.8%) fetuses with normal α-globin genes. Various α-thalassemia genotypes at different frequencies were found in the remaining 180 (54.2%) fetuses (Table 1). As shown in the table, in addition to normal fetuses, as many as 12 different α-thalassemia genotypes were encountered. The data indicated a diverse heterogeneity of α-thalassemia in our population. Among 180 fetuses with α-thalassemia, 57 (31.7%) were α-thalassemic fetuses with Hb Bart’s hydrops fetalis caused by homozygous α^0^-thalassemia (--/--). As this fatal thalassemic disease is one of the targets for prevention and control program and is associated with serious maternal complications, genetic counselling was given, and therapeutic abortion was offered in all cases. Other α-thalassemia genotypes encountered included α^0^-thalassemia trait (n = 44), α^+^-thalassemia trait (n = 26), homozygous Hb CS or compound heterozygous Hb CS/Hb Pakse’ (n = 14), Hb CS trait (n = 12), Hb H disease (n = 7), Hb H-CS or Hb H-Pakse’ disease (n = 6), double heterozygous for α^+^-thalassemia/Hb CS (n = 5), homozygous α^+^-thalassemia (n = 4), Hb H-Quong Sze (n = 1) and four unknown α-thalassemia. Further DNA sequencing of these four fetuses identified a double heterozygosity for Hb E and Hb Queens Park (α1 CD 32 A*T*G (Met) > A*A*G (Lys) in one fetus and a double heterozygosity for Hb E and Hb Amsterdam A1 (α1 CD 32 AT*G* (Met) > AT*A* (Ile) (data not shown), previously undescribed in the fetus (Fig. 3A, B). The two α-hemoglobinopathies could be confirmed using PCR–RFLP assay using *Bts*CI restriction enzyme as shown in Fig. 4. The α-thalassemia genotypes in two fetuses remained uncharacterized. As shown in Table 1, while Hb analysis of the fetal blood specimens detected no Hb Bart’s in the normal fetuses, variable amounts of Hb Bart’s were identified in the α-thalassemic fetuses. The amounts of Hb Bart’s corresponded with the severity of α-globin gene defects, i.e., highest in the Hb Bart’s hydrops fetalis (81.5 ± 3.6%) followed by Hb H disease, homozygous Hb CS, double α^+^-thalassemia/Hb CS, α^0^-thalassemia trait, and α^+^-thalassemia trait, as shown in Fig. 5. Interestingly, as high as 33.7% Hb Bart’s was detected in the fetus with Hb H-Quong Sze disease, who had severe anemia and developed hydrops fetalis. DNA analysis identified heterozygosity for α^0^-thalassemia in the father and Hb Quong Sze in the mother, as shown in Fig. 6.

**Table 1:** Fetal Hb Bart’s identified in each corresponding α-globin genotype

Item	Type	Number	α-Genotype	Hb Bart’s (%)
1	Non α-thalassemia	152	αα/αα	0
2	Hb Bart’s Hydrops fetalis	57	--/--	81.5 ± 3.6 (74.7–89.9)
3	α^0^-thalassemia trait	44	--/αα	4.8 ± 1.2 (1.9–8.0)
4	α^+^-thalassemia trait	26	-α/αα	0.7 ± 0.4 (0–1.5)
5	Homozygous Hb CS and Hb CS/Hb PS	14	α^CS^α/α^CS^α (11), α^CS^α/α^PS^α (3)	11.2 ± 2.6 (7.4–16.7)
6	Hb CS trait	12	α^CS^α/αα	1.3 ± 0.8 (0.4–2.9)
7	Hb H disease	7	--/-α	19.2 ± 1.5 (16.9–22.1)
8	Hb H-CS and Hb H-Pakse’ diseases	6	--/α^CS^α (5), --/α^PS^α (1)	26.3 ± 1.8 (23.4–28.2)
9	α^+^-thalassemia/Hb CS	5	α^CS^α/- α	7.3 ± 2.2 (4.8–9.9)
10	Homozygous α^+^-thalassemia	4	-α/-α	4.1 ± 1.5 (2.1–5.8)
11	Rare α^+^-thalassemia trait	2	αα^QP^/αα	0.5
			αα^Ams^/αα	0.5
12	Hb H-QZ disease	1	--/α^QZ^α	33.7
13	Unknown	2	na	0.7, 1.4
	Total	332		

Values are presented as mean ± SD (range)

*na* not available

CS = Hb Constant Spring, PS = Hb Pakse’, QP = Hb Queens Park, Ams = Hb Amsterdam A1, QZ = Hb Quong Sze

Prenatal characterization of the cases with fetal anemia and fetal cardiomegaly was done in two families (Figs. 1, 2). In family 1, the father was preliminarily diagnosed with Hb E heterozygote with 27.1% Hb E while the mother was a heterozygous Hb CS. Since the fetus developed fetal anemia and cardiomegaly of an unknown etiology, fetal blood was taken by cordocentesis at gestational age of 27 weeks for thalassemia investigation. Figure 1 listed hematological parameters of the fetus with Hb 5.1 g/dL and numerous nucleated RBC (750/100 WBC). Hb analysis identified Hb CS (3.1%), and Hb Bart’s (9.0%) in addition to the Hb F (81.9%) and a small amount of Hb A. DNA analysis revealed unexpectedly that the fetus was a homozygous Hb CS and identified the Hb CS gene in the father and the mother. Therefore, the father was in fact, a double heterozygote for Hb E/Hb CS, although Hb CS was not detected on Hb analysis. In family 2, according to the family history, the mother was a homozygous Hb E, and the father demonstrated a normal MCV value (85.9 fL). Unfortunately, their blood specimens were not available for further testing. As shown in Fig. 2, analysis of fetal blood taken at a gestational age of 34 weeks identified Hb 9.8 g/dL and nucleated RBC (225/100 WBC). Hb analysis revealed Hb E-CS (0.4%), Hb F-CS (1.2%), Hb Bart’s (13.6%) and Hb H (0.3%) in addition to the Hb F (80.3%), Hb E (1.0%) and a small peak of Hb A (3.2%). DNA analysis of α-globin gene identified that the fetus was a compound heterozygous for Hb CS/Hb Pakse’ with Hb E heterozygote.

## Discussion

Identifying α-thalassemia, especially α^0^-thalassemia is an essential step in a prevention and control program of severe thalassemia in Southeast Asia and China. Couples who both carry α^0^-thalassemia have a 25% risk of having offspring with Hb Bart’s hydrops fetalis caused by a homozygous α^0^-thalassemia, an emerging health care problem in many populations [17]. The disease is one of the targets of the prevention and control program of thalassemia in the region. In addition, the interaction of α^0^-thalassemia and α^+^-thalassemia leads to the Hb H disease, a thalassemia intermedia commonly encountered in the region. Some forms of Hb H disease, especially the non-deletional form, can lead to a severe phenotype and Hb H hydrops fetalis [18]. Accurate diagnosis of these α-thalassemia syndromes requires DNA analysis which could not be applied in the rural areas in the regions. The α-globin gene defects causing α-thalassemia result in excess of γ-globin chain (in the fetus) and β-globin chain (in adult), leading to the polymerization to form γ_4_ tetramer (Hb Bart’s) and β_4_ tetramer (Hb H). The appearance of Hb Bart’s and Hb H in peripheral blood are therefore good markers for α-thalassemia syndromes. In an adult, it is not difficult to detect Hb H in peripheral blood using either Hb H inclusion test or Hb-HPLC and capillary electrophoresis analysis [18, 19]. The Hb H inclusion test is relatively labor intensive and lacks sensitivity, especially in detecting double heterozygotes for α-thalassemia with Hb E and β-thalassemia because the β_4_ tetramer could be minimal [3–5]. It has been demonstrated that qualitative detection of Hb Bart’s in peripheral blood of adult subjects using immunochromatographic strip assay with anti-Hb Bart’s monoclonal antibody can help in the initial recognition of subject with α^0^-thalassemia although the test is not specific [20]. In the fetus and newborns, however, identification of Hb Bart’s is valid for detecting homozygous α^0^-thalassemia causing Hb Bart’s hydrops fetalis. In this most severe form of thalassemia, about 75% or more of Hb Bart’s, without Hb F and Hb A is usually observed [21–24]. This has been confirmed in Table 1, in which fetuses with Hb Bart’s hydrops fetalis (n = 57) had 81.5 ± 3.6% Hb Bart’s as measured by capillary electrophoresis. We found that an appearance of 75% or more Hb Bart’s in combination with no Hb F and Hb A is a definite diagnosis for Hb Bart’s hydrops fetalis with 100% accuracy, and α-globin genotyping by DNA analysis is not necessary.

The usefulness of quantification of fetal Hb Bart’s in prenatal diagnosis of severe α-thalassemia disease has been described. Unfortunately, in a previous preliminary study of Srivorakun et al. [21], fetal blood analysis was performed only on four α-thalassemia genotypes with relatively small numbers of samples i.e., homozygous α^0^-thalassemia (n = 4), Hb H disease (n = 3), α^0^-thalassemia carrier (n = 11) and α^+^-thalassemia carrier (n = 3). Non deletional α-thalassemia and many other α-thalassemia genotypes were not examined. Accordingly, the exact reference level of Hb Bart’s of each α-thalassemia genotype could not be ascertained. In the current study, as many as 12 α-thalassemia genotypes including both common and rare, and deletional and non-deletional types with higher numbers of subjects were studied, as listed in Table 1. Therefore, the expression level of Hb Bart’s for each α-thalassemia genotype could be reliably determined. Interestingly, as shown in Table 1 and Fig. 5, the amount of Hb Bart’s varied with the severity of the α-thalassemia syndromes. Thanks to the Hb-capillary electrophoresis, which can accurately report the amount of Hb Bart’s. The levels of Hb Bart’s were 33.7% in Hb H-Quong Sze disease, 26.3 ± 1.8% in Hb H-CS and Hb H-Pakse’ diseases and 19.2 ± 1.5% in Hb H disease. Although the fetus with Hb H-Quong Sze disease is reported here for the first time in Thailand, it has been shown in a Chinese patient that the disease was associated with severe anemia and Hb H hydrops fetalis [25]. Further reduction of Hb Bart’s to 11.2 ± 2.6% was noted in homozygous Hb CS and compound Hb CS/Hb Pakse’ disease (n = 14, Table 1), indicating a more excess γ-globin chain as compared to the α^0^-thalassemia trait (4.8 ± 1.2%) and homozygous α^+^-thalassemia (4.1 ± 1.5%). In this Hb CS disease, many fetuses have been associated with hydropic change suggesting fetal anemia, requiring intrauterine transfusion for effective management [8, 9, 26]. Early diagnosis of the case is therefore essential. Examples are illustrated in Fig. 1 (homozygous Hb CS) and Fig. 2 (compound heterozygote for Hb CS/Hb Pakse’ with Hb E). The latter has been reported herein for the first time in the fetus. Both fetuses were associated with fetal anemia and cardiomegaly. It is noteworthy that patient 1 had lower Hb and Hct values than patient 2. This is likely due to the difference in gestational ages at which the fetal blood samples were analyzed. These were 27 and 34 weeks for patients 1 and 2, respectively. It has been shown that hematological parameters varied with gestational age. An increase in gestational age is associated with increased Hb and Hct and decreased MCV, MCH, and MCHC values [27–29]. In addition, a co-inheritance of the Hb E, which is a β-globin chain variant, in patient 2 may help in the improvement of the hematological phenotype of the patient. Hb analysis of the fetal blood samples identified Hb Bart’s 9.0% and 13.6%, respectively. As in the Hb CS trait, the level of Hb Bart’s was 1.3 ± 0.8% (Table 1), the average level of Hb Bart’s at 11.2 ± 2.6% is a useful diagnostic marker for a homozygous Hb CS and a compound Hb CS/Hb Pakse’ diseases. Prenatal diagnosis of the diseases is recommended so that earlier and appropriate management can be given.

In our study, two rare α-thalassemia variants were encountered for the first time in the fetuses i.e. the Hb Queens Park (HBA1:c.98T>A) [α32(B13)Met → Lys] (Fig. 3A) and Hb Amsterdam A1 (HBA1:c.99G>A)[α32(B13)Met → Ile] (Fig. 3B). The latter was found for the first time in association with Hb E heterozygote. The levels of Hb Bart’s at 0.5% in both fetuses confirmed that these two α-thalassemia variants are mild forms of α^+^-thalassemia. Hb Queens Park has been reported originally in a patient of Western Australia with a mild thalassemia phenotype [30]. Association of Hb Queens Park with α^0^-thalassemia (SEA deletion) led to the non-deletional Hb H disease in a Thai boy with a mild clinical phenotype comparable to that of the common deletional Hb H disease [31]. Herein described is for the first time Hb Queens Park was identified in association with Hb E in the fetus and with α^0^-thalassemia (SEA deletion) and Hb E trait, leading to the Hb Queens Park AEBart’s disease in the mother who demonstrated a thalassemia intermedia phenotype (Fig. 3A). Hb Amsterdam A1 is a rare hyperunstable α chain variant due to perturbation of globin-heme and α1β1 subunit interactions [32]. Identifying a Thai fetus with double heterozygote for Hb E/Hb Amsterdam A1 is the second report of this rare α-thalassemia variant ever documented in the literature. Since no abnormal Hb band corresponding to the Hb Amsterdam A1 was visible on alkaline electrophoresis, high performance liquid chromatography (HPLC), and capillary electrophoresis, screening for this α-thalassemia variant should be done by PCR–RFLP assay using *BtsC*1 restriction enzyme as shown in Fig. 4. This method could identify all the three α-thalassemia mutations found in the region namely Hb Queens Park, Hb Amsterdam A1, and IVS1-117 (G-A), recently described in a Laos patient [6].

In conclusion, our results demonstrated that analysis of fetal Hb using capillary electrophoresis could be an effective alternative to DNA characterization of fetal α-thalassemia syndromes. In fact, any method of Hb analysis should be applicable, provided the quantitative identification of Hb Bart’s is accurately reported. Our results in Table 1 and the previous study using anti-Hb Bart’s monoclonal antibody [20] indicate likely that fetal red blood cells of all α-thalassemia syndromes found in the region contain adequate amounts of Hb Bart’s for accurate quantification. Although, as compared to the chorionic villi sampling and amniocentesis, cordocentesis to obtain fetal blood specimen is not a common practice in prenatal diagnosis, it is still helpful for late pregnancy and for those of at-risk couples whose mutations are not characterized [12]. The fetal blood analysis and quantification of Hb Bart’s using capillary electrophoresis which is simple, rapid, fully automated, and applicable in most routine prenatal diagnosis laboratories should facilitate a prevention and control program for severe thalassemia diseases in the region.

## Data Availability

Please contact corresponding author for data request.

## Declarations

### Completing interests

The authors declare that they have no completing interests.
